# How do Chinese students effectively convey emotions through the discussion forums in the LMOOCs?

**DOI:** 10.3389/fpsyg.2023.1128089

**Published:** 2023-02-17

**Authors:** Binfeng Chen, Xinmin Zheng

**Affiliations:** ^1^School of Foreign Languages, Fujian Normal University, Fuzhou, Fujian, China; ^2^Department of Foreign Languages, College of Zhicheng, Fuzhou University, Fuzhou, China; ^3^School of Education, Shanghai International Studies University, Shanghai, China

**Keywords:** LMOOCs, sentiment analysis, content analysis, discussion forum, interaction, data collection, data analysis

## 1. Introduction

Massive Open Online Courses (MOOCs) have received continuing academic attention in recent years due to their significant impact on higher education (Pappano, 2012; Galán et al., 2019). Among them, language MOOCs (LMOOCs) have now become an emerging research interest in the field (Bárcena and Martín-Monje, 2014). Plentiful empirical studies have generated abundant findings, which enable us to better understand the benefits and limitations (e.g., Peral, 2019; Bartalesi-Graf et al., 2022) as well as the teaching models and design (e.g., Appel and Pujolà, 2021; Luo and Ye, 2021). However, crucial as emotions are in the learning context (Rowe et al., 2015; Gil-Madrona, 2020), to date few empirical studies have been conducted to closely examine the latent emotions of LMOOC students. Particularly in the post-pandemic years with the surging popularity of online courses, further research is badly needed (Chacón-Beltrán and Echitchi, 2021).

Recently, Peng and Jiang (2022) published their article entitled *Mining opinions on LMOOCs: Sentiment and content analyses of Chinese students' comments in discussion forums* (hereinafter it is referred to as “the article”), the first sentiment and content analyses of comments in the LMOOC discussion forums. This study has contributed timely responses to the growing body of research. Specifically, by investigating the comments of the 60 LMOOCs in China, the article reveals that the majority of comments examined are positive, and five major themes are summarized and examined closely to probe into students' subtle emotions and attitudes. As far as we are concerned, the article is rigorous and convincing, which enables us to better understand the emotions and opinions of Chinese LMOOC students and beyond. Illuminating and Inspirational as it is, there seems to be room for further consideration. Thus, we would like to comment on this article to evaluate online engagement and web-based course design for the purpose of promoting more future relevant research studies.

Before starting the analysis and discussion, we find it necessary to review two key concepts in the article, i.e., sentiment analysis and content analysis, to help the reader better understand the article. First, sentiment analysis, or opinion mining, is a type of natural language processing that involves the extraction of people's opinions of and sentiments toward given topics, products, issues, and persons (Liu, 2020). Sentiment analysis includes such tasks as aspect and entity extraction, sentiment classification, sentiment summarization and search (Li and Hovy, 2015), and typically it examines the polarity and strength of the sentiment in terms of its positive, neutral, or negative nature (Poria et al., 2018), and this can be carried out at the document, sentence or aspect level (Liu, 2020). Although the aspect extraction is crucial (Tubishat et al., 2018), sentence and document analyses are also useful (Birjali et al., 2021). Therefore, more often than not, two or more levels of analyses are conducted to achieve better performance (Mai and Le, 2021). Sentiment analysis, with its great theoretical and practical value, is widely applied to mine opinions, analyze emotions and extract information from social media and beyond. However, there are still some challenges for sentiment analysis such as sarcasm detection (Ravi and Ravi, 2017) and implicit aspect extraction (Birjali et al., 2021).

Second, content analysis is a research method to make systematic and reliable interpretations of the content of text data (Drisko and Maschi, 2015). Three major trends can be identified in content analysis. To begin with, contemporary content analysis tends to go far beyond the *manifest content*, or the surface structure of the data (Berg and Lune, 2016) that is explicit or easily noticeable, and focuses on the *latent content*, or implied information hidden in the text, ranging from the cognitive judgments to emotional feelings (Riff et al., 2005). Furthermore, content analysis can be conducted both qualitatively and quantitatively. While quantitatively oriented content analysis emphasizes reliability, validity and objectivity, qualitatively oriented content analysis focuses on replicability, validity and transparency (Drisko and Maschi, 2015). As Lacy et al. (2015) pointed out, issues such as sampling and reliability may pose great challenges to content analysis, and we should be especially careful in handling them. In addition, content analysis can be combined with other types of analysis to generate an integrated methodology (Neuendorf, 2012), as contemporary content analysis faces a wider context with larger volumes of data (Krippendorff, 2018).

To the best of our knowledge, the article combines sentiment analysis with content analysis to examine student comments in the discussion forums in major Chinese LMOOCs, which arouses our keen interest in making comments. To guide our study, we raise two research questions as follows:

RQ 1: To what extent is the data collection suitable for this study?
RQ 2: In what way did the article analyze the data to address the research questions raised?

To answer the above two research questions, we adopt the embedded analysis method (Creswell and Poth, 2018), which serves as an important tool to focus on a specific question. Instead of analyzing the article holistically, we mainly concentrate on the key issues and specific aspects of the article, which has the advantage of examining the particular phenomenon in operational detail (Yin, 2009). Thus, guided by the two research questions, we are going to focus on the data collection and data analysis within the article, and then spot common and divergent opinions that transcend the article. In the following sections, we first briefly summarize the article, then discuss its pros and cons, and finally conclude with implications and suggestions for further studies.

## 2. The study

Aiming to explore Chinese students' opinions and sentiments in online learning, the article conducted sentiment and content analyses to examine Chinese students' comments in LMOOC discussion forums. By adopting both quantitative and qualitative methods, the article investigates Chinese students' perceptions and needs regarding LMOOC learning.

Specifically, with regard to data collection, the article selected student comments from 60 LMOOCs in China based on the existing research project covering 30 high-quality LMOOCs accredited by the Ministry of Education and 30 regular ones. Of the 60 LMOOCs, 56 were provided on iCourse, three on XuetangX, and one on Treenity, and all 60 were related to English for general or specific purposes. These courses were typically xMOOCs, which are mainly syllabus-based (Hew et al., 2018) while interactions are not highly expected (Motzo and Proudfoot, 2017). Student comments on these courses were gathered in the time span from September 2020 to March 2021. After the data cleansing by removing emoticons and other irrelevant information, altogether 22,368 comment entries were obtained for analysis.

Concerning data analysis, the article adopted sentiment and content analyses to answer the four research questions. First, with the software ROST CM6, the article computed the sentiment scores of each LMOOC, including the scores of each comment entry, of each student and of all the students, to identify the distribution of student sentiment (i.e., positive, neutral, and negative) in their comments. Second, with the combination of the Python package SnowNLP and IBM SPSS Statistics V22, the article examined the correlation between student sentiment and course rating to test the validity of the analytical results. To complement the above two steps of sentiment analysis and further investigate latent opinions in student comments, the article next conducted two steps of content analysis. Third, the article used JMP Pro 16 software to further identify the major themes of students' comments in clusters through rounds of observations. Last, assisted by NVivo 12, content analysis of latent concerns and opinions from the LMOOC students was conducted to obtain empirical evidence to inform LMOOC implementation. Thus, different research tools were used to progressively probe into students' sentiments and concerns.

Regarding the major findings, first, it was revealed that Chinese students' online comments were predominantly positive, that is, 20,988 out of 22,368. Furthermore, the positive correlation between student sentiment and course rating demonstrated that students with positive sentiments were more likely to rate higher for the course. Following that, the article spotted five major themes (i.e., attitudes toward the LMOOCs, comments on the LMOOCs, evaluation of LMOOC instruction and instructors, learning outcomes, and suggestions), and further frequency counts of the codes also revealed that the majority of the codes were positive, consistent with the above sentiment analysis. Moreover, the article identified four categories of students' negative comments (i.e., course content, lecture videos, assignments and tests, and platforms) and five types of students' suggestions (i.e., suggestions for instructors, for LMOOC design, for video design, for assignments and tests, for platforms). Intriguingly, it was discerned that students might use euphemistic expressions and buzz words to circumvent the negative feedback, which could possibly be explained by the mandatory nature of the discussion forum for students. Overall, the study has implications for LMOOC teachers to improve their online instruction and for LMOOC developers to update the course design, particularly in relation to the means of tailoring the course content and teaching styles to students' needs.

## 3. Discussion

### 3.1. Data collection

Regarding data collection, the article analyzed the comment entries in the discussion forums in 60 LMOOCs in China to mine students' sentiments and opinions. On the one hand, the data collected by the article are basically inclusive and valid and serve the research purpose well. To enhance the reliability and validity of the study, the article, after two rounds of data cleansing, collected inclusively 22,368 comments for analysis from 60 LMOOCs, which covers 30 high-quality ones and 30 regular ones. According to Creswell and Poth (2018), sample size is important for the reliability and validity of studies, and it is well established that larger sample sizes tend to be more stable (Kretzschmar and Gignac, 2019). Thus, by taking different variables into consideration, we believe that the data collected in the article are basically reliable and valid.

On the other hand, there seems to be room to refine the data so as to better address the research questions. Firstly, it remains unclear as to why the courses under analysis are delivered mainly in English and 56 courses out of 60 are taken from iCourse. This, in our opinion, can possibly be attributed to the relatively easy access to the data by analyzing English MOOCs on iCourse. As China boasts the largest number of English learners in the world (He and Li, 2021) and iCourse is the largest MOOC platform in China (Wu and Chen, 2021), the choice of an overwhelming number of English MOOCs from iCourse would make data collection more easily accessible and feasible. However, this lack of data diversity could affect its reliability, as data heterogeneity is integral to the validity of analysis (Pitard, 2019). Therefore, it is necessary to diversify the data sources to ensure the depth and width of the data as far as possible (Kretzschmar and Gignac, 2019). In fact, as languages other than English are not compulsory in China, MOOCs of other languages might cater mainly to beginners and interactions seem almost inevitable (Furnborough, 2012). Thus, for a better understanding of the LMOOCs at large, the inclusion of LMOOCs other than English for analysis might bring forth intriguing results in future studies. Besides, to ensure the inclusion of the most representative LMOOCs in the study (Lohr, 2022), we suggest that diverse platforms (e.g., UMOOCs, a platform devoted to language learning) be taken into consideration. In this way, it is possible to balance the comprehensiveness and representativeness of the data so as to enhance its reliability and validity.

Secondly, we doubt the selection of course types in the article, as all the courses under scrutiny fell into the category of xMOOCs. As is known to us, xMOOCs are typically lacking in interactions (Motzo and Proudfoot, 2017). However, as LMOOCs are skill-based and socially-oriented in nature (Martín-Monje et al., 2018; Bartalesi-Graf et al., 2022), the choice of xMOOCs as the single type of courses for analysis might not fit the interaction-based research perfectly. Therefore, for the sake of comprehensiveness, we think some other types of LMOOCs should be selected in future studies to better answer the research questions. For instance, cMOOCs, which are based on a connectivist approach with emphasis on social learning and learner autonomy (Downes, 2012), might be suitable for this study to probe into student sentiment in the course of interactions. Hence, we hold that the hybrid use of xMOOC-type and cMOOC-type LMOOCs for analysis is essential (García-Peñalvo et al., 2017) and would more effectively and systematically resolve the research questions.

Thirdly, we are also skeptical of the use of student comments in the discussion forums as the only source of data. As student engagement patterns in LMOOCs vary (Martín-Monje et al., 2018), the channels for students to express feelings also differ. Among all these channels, previous studies have shown that online comments may not truly reflect their genuine feelings (Chen and Pain, 2017; Kim et al., 2021), partly because students tend to include some necessary and sufficient information about themselves in MOOCs (Zubkov and Morozova, 2017), and partly because the mandatory nature of the comments could leave students' passive or even irrelevant comments unavoidable (Peng and Jiang, 2022). Therefore, to better understand students' psychological states such as emotions and motivations in the course of online engagement (Fririksdóttir, 2021; Wright and Furneaux, 2021), the sole use of the comment in the discussion forum may not be sufficient. As far as we are concerned, student comments on Weibo (the Chinese equivalent of Twitter), an encompassing platform for expressing views freely, are the ideal supplementary data sources for sentiment analysis of online courses (Zhou, 2020) to enhance the reliability of data.

All in all, given the great importance of data collection (Sapsford and Jupp, 2006), we appeal that future studies should give weight to the design before collection. As far as we are concerned, multiple factors should be taken into account in the course of data collection for the sake of data saturation (Merriam and Tisdell, 2015). Therefore, if possible, we may diversify the data sources and enrich the types of data to ensure the reliability and validity of the research (Kretzschmar and Gignac, 2019).

### 3.2. Data analysis

Concerning data analysis, the article used sentiment and content analyses with an array of tools to reveal the sentiment polarity and identify key themes and sentiments of the comments. In general, we find the sentiment analysis relatively thorough and convincing, with student comments scrutinized and student emotions mined step by step. Moreover, we also notice that content analysis was used in the article to complement sentiment analysis to enhance validity (Neuendorf, 2012; Drisko and Maschi, 2015). However, we hold that there is still room to dig deeper for future studies.

First, the analysis of the article centered around the emotional polarity of student comment, which is a typical way of sentiment analysis (Poria et al., 2018). However, as is stated above, students' online engagement involves cognitive, emotional, and behavioral dimensions, and is a complex process challenging to pin down (Riff et al., 2005). For Evans (2002), emotions may also be cultural, anthropological or even scientific, which makes it elusive to capture. Therefore, for an in-depth data analysis we believe it is necessary to transcend the traditional positive-negative dichotomy of sentiment polarity and probe into the diverse variables and subtle meanings in their sentiments and opinions. In fact, while Poria et al. (2018) proposed multimodal sentiment analysis, Belli (2018) applied this multimodal approach to analyze how participants manage their negative emotions during online learning, both of which provide us with possible ways to consider different variables of emotions and detect their heterogeneity (Gil-Madrona and Díaz, 2012; Gil-Madrona and Martínez, 2016). Besides, to warn against the danger of “dark glasses” which might hide our true emotions (Anolli et al., 2002), we should be particularly careful in the detection of some ironical or euphemistic expressions and the implicit aspect in the comments (Ravi and Ravi, 2017; Birjali et al., 2021). This disguise of genuine feelings in student comments also brings forth another interesting topic for further research, that is, emotion management, which is largely under-examined in the learning context (Roberts and Smith, 2002; Goodwin, 2007; Garner, 2010; Belli, 2018). Furthermore, to capture the subtleties of opinions more accurately, we might adopt slightly different sentiment analysis tools and models for different discussion forums, i.e., prescribed discussions, free discussions and Q&A discussions (Yang et al., 2015). For instance, Mukherjee and Liu (2012). JTE Model may be more appropriate for free discussions, while behavioral, graph or probabilistic models might be needed to detect fake or deceptive opinions in the comments (Liu, 2020).

Second, we appreciate that the article analyzed the aspect-level and sentence-level sentiments, since the two levels of analysis together might yield more accurate results and achieve better performance (Mai and Le, 2021). Nevertheless, as sentiment analysis can be carried out at the document, sentence or aspect level (Liu, 2020), it can possibly go further to include the document-level sentiment analysis. Some other scholars also proposed concept-level analysis to complement current domain-dependent sentiment analysis (Bisio et al., 2017). As a matter of fact, the article has eliminated such important information as emoticons from analysis, which makes the document analysis imcomplete. As far as we are concerned, multi-level sentiment analysis can help detect vague semantic links and complicated sentiment information (Ha et al., 2019), hence it is of great significance for comment analysis. Besides, as forum comments abound in extremely short comments with ineffective or irrelevant information (Babori, 2021), further document-level analysis may help cleanse the data noise and enhance its reliability. Therefore, we maintain that more comprehensive further studies can be carried out to detect the subtly different sentiments at different levels.

To sum up, there are many potential productive avenues for future research on the delicate emotional intensities and latent meanings of the comments. On the one hand, we suggest it is necessary to probe further to transcend the positive-negative dichotomy of sentiment polarity and capture the subtle sentiments and opinions in the comments. On the other hand, we suggest further research studies should broaden the scopes of the analysis to get a comprehensive understanding of student sentiments.

## 4. Conclusion

To summarize, this is generally an in-depth study of the general sentiments and opinions in LMOOC comments by adopting both quantitative and qualitative analysis tools. It reveals that student comments are dominantly positive and can be categorized into five sets of themes. As the first empirical study to investigate students' emotions toward and views of LMOOCs with the combination of sentiment analysis and content analysis, the article has successfully resolved the four research questions, illuminating later studies and future practice in this field. The article might shed light on the future LMOOC design to tailor students' needs, and illuminate further studies to investigate multi-level student sentiments in various contexts. Therefore, we would highly recommend it to scholars interested in the sentiment analysis of LMOOCs, and call for more comprehensive and in-depth exploration in future research.

## Author contributions

BC drafted the initial manuscript. XZ helped him select the commented article, provided insights and suggestions during the process of writing and revised the last draft. Both authors contributed to the article and approved the submitted version.
